# Estimating Body Composition in Adolescent Sprint Athletes: Comparison of Different Methods in a 3 Years Longitudinal Design

**DOI:** 10.1371/journal.pone.0136788

**Published:** 2015-08-28

**Authors:** Dirk Aerenhouts, Peter Clarys, Jan Taeymans, Jelle Van Cauwenberg

**Affiliations:** 1 Department of Human Biometry and Biomechanics, Faculty of Physical Education and Physical Therapy, Vrije Universiteit Brussel, Brussels, Belgium; 2 Department of Physiotherapy, Faculty of Medicine and Health Sciences, University of Antwerp, Antwerp, Belgium; 3 Department of Health, Bern University of Applied Sciences, Bern, Switzerland; 4 Department of Public Health, Faculty of Medicine and Health Sciences, Ghent University, Ghent, Belgium; 5 Fund for Scientific Research Flanders, Brussels, Belgium; Vanderbilt University, UNITED STATES

## Abstract

A recommended field method to assess body composition in adolescent sprint athletes is currently lacking. Existing methods developed for non-athletic adolescents were not longitudinally validated and do not take maturation status into account. This longitudinal study compared two field methods, i.e., a Bio Impedance Analysis (BIA) and a skinfold based equation, with underwater densitometry to track body fat percentage relative to years from age at peak height velocity in adolescent sprint athletes. In this study, adolescent sprint athletes (34 girls, 35 boys) were measured every 6 months during 3 years (age at start = 14.8 ± 1.5yrs in girls and 14.7 ± 1.9yrs in boys). Body fat percentage was estimated in 3 different ways: 1) using BIA with the TANITA TBF 410; 2) using a skinfold based equation; 3) using underwater densitometry which was considered as the reference method. Height for age since birth was used to estimate age at peak height velocity. Cross-sectional analyses were performed using repeated measures ANOVA and Pearson correlations between measurement methods at each occasion. Data were analyzed longitudinally using a multilevel cross-classified model with the PROC Mixed procedure. In boys, compared to underwater densitometry, the skinfold based formula revealed comparable values for body fatness during the study period whereas BIA showed a different pattern leading to an overestimation of body fatness starting from 4 years after age at peak height velocity. In girls, both the skinfold based formula and BIA overestimated body fatness across the whole range of years from peak height velocity. The skinfold based method appears to give an acceptable estimation of body composition during growth as compared to underwater densitometry in male adolescent sprinters. In girls, caution is warranted when interpreting estimations of body fatness by both BIA and a skinfold based formula since both methods tend to give an overestimation.

## Introduction

Early specialization and the demands concerning performance level reinforce the need for a sport specific body size and composition already at young age. When striving for a specific morphology and to balance training load, it is highly important to accurately follow up the adolescent athlete’s body composition. The risk of a false result of body composition assessment in athletes is that they may unnecessarily adapt their nutritional and training strategies, causing energy availability deficiency and an evolution towards a less suited body composition with a negative impact on health and performance [1].

Adult sprint athletes are recognized as strength athletes who need to achieve a high power to weight ratio by maximizing muscle mass and maintaining low body fat [2]. It should be recognized that the adolescent athlete has a different body composition compared to adult athletes. Moreover, adolescent growth is characterized by rapid changes in fat and components of fat free mass, body components that are known to evolve in a sex specific manner. Often, the adolescent growth spurt is preceded by a rapid increase in body fatness, also known as the ‘pre-pubertal fat wave’ [3]. About 5 months to a year after peak height velocity (PHV), a rapid increase in fat free mass can be observed, especially in boys [4]. However, a high variability in timing and tempo of biological maturation can be observed between individuals of the same sex resulting in early and late maturers [3,4]. Consequently, methods used to estimate body composition in adolescents should not only be sex specific, but also sensitive to biological maturation. To track the maturation process in adolescents, it is useful to follow up linear growth for which age at PHV is a useful landmark.

While highly accurate laboratory techniques exist, field methods remain important to determine body composition especially for promising youngsters who often do not have access to the most modern but often expensive techniques.

Despite well-known drawbacks concerning accuracy and sensitivity to hydration status of the subject [5], underwater weighing densitometry (UWD) remains a more accessible and affordable method to estimate body composition in athletes as compared to medical laboratory techniques. Indeed, UWD can still be regarded as the preferred two-component method on the condition that standardized procedures are respected [5,6].

Bio-impedance analysis (BIA) and anthropometric based formulae are widespread field methods. Numerous equations for both skinfolds (SF) and BIA techniques have been developed, all with a limited population specific applicability [5]. To assess body composition in a specific type of athlete, selecting the most appropriate field technique, with the most appropriate formula, remains challenging. Indeed, for adolescent sprint athletes, no clear recommendation for a field method to assess body composition exists to date. According to the review of Norgan et al. [5], the SF formula of Slaughter et al. [7] is recommended for estimating body composition in adolescents. However, validation of this and other SF and BIA equations was done using cross-sectional designs. Therefore, the question remains whether these techniques are suitable for longitudinal use (follow-up). Moreover, the age range for which existing formulae were developed is based on calendar age, not taking into account the maturity status of the subject. This is a problem considering the high variability in maturity observed in adolescents of the same age and sex [3,4]. Thus, the accuracy of the current field methods during the critical years of adolescence has not been investigated. To investigate the applicability of different body composition assessment field methods in adolescent sprint athletes a longitudinal study design that accounts for maturity status, is warranted.

This longitudinal study aimed to compare BIA and the SF based equation of Slaughter et al. [7] (index tests) with underwater densitometry (reference test) as methods to track body composition relative to years from age at PHV in adolescent sprint athletes.

## Methods

### Participants

In cooperation with the Flemish Athletics League, 120 athletes aged 12 to 18 years were selected and invited to participate in a follow-up study on nutrition, anthropometric characteristics, and performance. The selection was based on in- and outdoor sprint discipline rankings, including all top ten ranked athletes competing in distances from 60 to 400 m flat and hurdles. A total of 34 girls and 35 boys accepted the invitation to participate. Age at start was 14.8 ± 1.5 years in girls and 14.7 ± 1.9 years in boys. They were competing in their discipline for at least two years. All athletes were involved in a sprint training program of three or more training bouts per week.

### Ethics Statement

In accordance with the declaration of Helsinki, all participating athletes were given detailed information about the study and were asked to give their written informed consent in the presence of their parents. The study and the consent procedure were approved by the Vrije Universiteit Brussel Medical Ethical Committee.

### Measurements

From the autumn of 2006 until the spring of 2008, body fatness was estimated bi-annually using BIA, SF and UWD. Autumn measurements were carried out during November and December whilst spring measurements were carried out during May and June, respecting as close as possible the six months interval for every subject.

All anthropometric data were collected according to the International Society for the Advancement of Kinanthropometry guidelines [8]. Body height was measured with a wall mounted stadiometer to the nearest 0.1 cm. Skinfold thickness data were collected with a calibrated Harpenden caliper (Baty International, West Sussex, UK) accurate up to 0.2 mm. In order to avoid errors, measurements of skinfolds were repeated two to three times with a maximum allowed deviation of 5%.

The TANITA TBF 410 (Tanita Corp., Tokyo, Japan) was used to measure body weight to the nearest 100 g as well as to estimate body fat percentage using BIA. For these purposes, athletes were measured in their underwear for which a correction in body weight was made by the device. For the BIA, standardized procedures were followed, based on the guidelines described by Kushner et al. [9]. Furthermore, athletes removed all conductive material such as watches and jewelry. Body fat percentage was estimated by selecting the ‘athletic’ formula including the foot to foot current resistance, body height, weight and age. The exact prediction formula was not provided by the manufacturer.

Secondly, body fat percentage was estimated with the formula of Slaughter et al. [7]:

$$Boys\;11–14\;years:\;body\;fat\;\%=1.21\;(T+S)–0.008\;{\left(T+S\right)}^{2}–3.4$$

$$Boys\;14.1–20\;years:\;body\;fat\;\%=1.21\;(T+S)–0.008\;{\left(T+S\right)}^{2}–5.5$$

$$Girls\;11–20\;years:\;body\;fat\;\%=1.33\;(T+S)–0.013\;{\left(T+S\right)}^{2}–2.5$$

where (T+S) stands for the sum of the triceps and subscapular skinfolds.

This formula is recommended to use in adolescents in the review by Norgan et al. [5].

Finally, as the standard method (reference test), body density was measured through underwater weighing and body fat percentage was calculated with the formula of Siri [10]:

$$Body\;fat\;\%=\left(\frac{495}{bodydensity}\right)–450$$

withbody density calculated using the following formula:

$$body\;density=Wt/[\left(Wt–\frac{Ww}{dw}\right)–(RV+GI)]$$

where Wt is body weight in air, Ww is body weight in water accurate to 1 g (highest value of 5 measurements, after maximal expiration), dw is the density of the water (determined after measuring the water temperature), RV is residual lung volume (based on gender, height, and weight), and GI is the gas in the gastrointestinal tract (fixed to 100 g).

### Age at peak height velocity

Height for age data from birth until the start of the study, collected by governmental childcare organizations, were obtained via the parents. In Belgium, these data are collected with an interval of 1 to 2 months during the first 2 years after birth, and thereafter about once every year. In combination with height for age data collected during the present study, the growth curve and age at peak height velocity (PHV) was obtained. For this purpose, the Jolicoeur-Pontier-Abidi-2-method was applied using the Analysis of Growth Curves software program [11]. Years from PHV was calculated on every measurement occasion by subtracting age at PHV from calendar age.

### Data analysis

All analyses were performed separately for boys and girls and analyzed cross-sectionally as well as longitudinally. Cross-sectional analyses were performed using SPSS (version 22.0, IBM Corp., NY) whilst longitudinal analysis in function of years from PHV was performed in SAS (version 9.3, SAS Institute, Inc.) using multilevel modelling.

The Kolmogorov-Smirnov Goodness of Fit Test indicated that all variables were normally distributed.

#### Drop-out analysis

Drop-out analysis was performed by comparing baseline values of age, body height, body weight and body fat percentage as estimated by UWD between athletes who were measured on each occasion and those athletes with at least one missing value. For this purpose an independent samples t-test was applied.

#### Cross-sectional analysis

For the cross-sectional analysis, a one-way repeated measures ANOVA was applied to compare the three methods at each occasion. When a significant difference was found, a paired samples t-test with Bonferroni correction was used to locate the difference. Pearson correlations were calculated between the different methods at each occasion.

#### Longitudinal analysis

For the longitudinal analysis, multilevel cross-classified models were fitted with repeated measurements clustered within participants and repeated measurements clustered within measurement methods using the PROC Mixed Procedure. Cross-classified models were used since measurements were nested within the cells of the two-way cross-classification of participants by measurement methods [12,13].

The pattern of body composition according to years from PHV and the three measurement methods was examined by analyzing interaction effects between years from PHV and measurement method. The models were adjusted for body height and weight. Before fitting a final model, the best fitting residual covariance structure was searched based on Akaike’s Information Criterion. For boys, a heterogeneous compound symmetry covariance structure was selected. For girls, a heterogeneous autoregressive covariance structure yielded the best fit. All models were estimated using restricted maximum likelihood [13]. The final model was used to predict body fat percentages according to years from PHV and measurement method. Finally, p-values were calculated to examine the differences in body fat percentages between the three measurement methods at different years from PHV.

Significance level for both the cross-sectional and longitudinal analysis was determined at 0.05.

## Results

### Drop-out analyses

During the entire study period a high drop-out of subjects was observed. Only in 38% of all participating adolescent athletes body composition could be estimated using the three different methods on each occasion. These athletes did not differ from subjects with at least one missing value in baseline values of age, body height, body weight and body fat percentage. A cessation of the athletic career and injuries were the main reasons for drop out or missing data. However, the PROC Mixed procedure that was used for the longitudinal analysis does not delete missing data list wise, implying that participants with missing measurements were not excluded from longitudinal analysis and all available observations were used. Consequently, 359 observations in boys (57%) and 371 observations in girls (61%) were included in the longitudinal analysis.

### Cross-sectional results

Cross-sectional results for body fat percentage according the three different methods, are presented in Tables 1 and 2, for boys and girls respectively. Also presented in Tables 1 and 2 are the correlations between UWD on the one hand and BIA and SF on the other.

**Table 1:** Pearson correlation with UWD (between brackets) and cross sectional comparison of body fat % (mean ± SE) in boys estimated by UWD, BIA and SF.

Occasion	Age (year)	Fat % UWD	Fat % BIA (r with UWD)	Fat % SF (r with UWD)
**1 (n = 33)**	14.7 ± 0.3	10.2 ± 0.7 ^a^	9.7 ± 0.5 ^a^ **(.46)**	9.5 ± 0.4 ^a^ **(.43)**
**2 (n = 32)**	15.2 ± 0.3	9.3 ± 0.7 ^a^	9.7 ± 0.6 ^a^ **(.51)**	9.4 ± 0.4 ^a^ **(.46)**
**3 (n = 29)**	15.7 ± 0.4	9.4 ± 0.4 ^a^^,^^b^	7.9 ± 0.6 ^a^ (.14)	9.4 ± 0.4 ^b^ **(.45)**
**4 (n = 27)**	16.1 ± 0.4	8.1 ± 0.6 ^a^^,^^b^	7.1 ± 0.5 ^a^ (.25)	8.5 ± 0.3 ^b^ **(.55)**
**5 (n = 26)**	16.7 ± 0.4	8.4 ± 0.6 ^a^^,^^b^	6.4 ± 0.6 ^a^ (.10)	8.6 ± 0.3 ^b^ (.32)
**6 (n = 22)**	17.2 ± 0.4	8.0 ± 0.4 ^a^^,^^b^	6.7 ± 0.5 ^a^ (-.04)	9.1 ± 0.4 ^b^ **(.64)**

UWD: Underwater Densitometry; BIA: Bio Impedance Analysis, SF: Skinfold formula of Slaughter et al. (1988)

^a,b^ Within one row, means with the same indices measurement methods do not differ significantly (α = 0.05). Correlation in bold means significant with UWD (α = 0.05).

**Table 2:** Pearson correlation with UWD (between brackets) and cross sectional comparison of body fat % (mean ± SE) in girls estimated by UWD, BIA and SF.

Occasion	Age (year)	Fat % UWD	Fat % BIA (r with UWD)	Fat % SF (r with UWD)
**1 (n = 32)**	14.8 ± 0.3	18.4 ± 1.0 ^a^^,^^b^	18.6 ± 1.0 ^a^ (.30)	16.9 ± 0.7 ^b^ **(.38)**
**2 (n = 30)**	15.3 ± 0.3	17.0 ± 0.8 ^a^	20.7 ± 1.1 ^b^ **(.66)**	18.0 ± 0.7 ^a^ **(.77)**
**3 (n = 29)**	15.7 ± 0.3	16.4 ± 0.8 ^a^	18.3 ± 1.0 ^b^ **(.67)**	17.4 ± 0.7 ^a^^,^^b^ **(.71)**
**4 (n = 26)**	16.3 ± 0.3	16.5 ± 0.8 ^a^	19.0 ± 1.0 ^b^ **(.71)**	17.9 ± 0.8 ^b^ **(.77)**
**5 (n = 23)**	16.8 ± 0.3	18.6 ± 1.2 ^a^	16.5 ± 1.1 ^a^ (.36)	17.9 ± 0.8 ^a^ (.38)
**6 (n = 19)**	17.2 ± 0.4	16.3 ± 0.9 ^a^	18.1 ± 0.9 ^a^ **(.57)**	17.7 ± 0.8 ^a^ **(.58)**

UWD: Underwater Densitometry; BIA: Bio Impedance Analysis; SF: Skinfold formula of Slaughter et al. (1988)

^a,b^ Within one row, means with the same indices measurement methods do not differ significantly (α = 0.05). Correlation in bold means significant with UWD (α = 0.05).

In boys, BIA nor SF gave a different estimation of body fat percentage as compared to UWD. Estimations from the BIA and SF method differed significantly from each other on occasions 3 until 6.

In girls, the BIA method gave a significant overestimation of body fatness as compared to UWD on the 2^nd^, 3^rd^ and 4^th^ occasion. The SF based estimation was comparable to UWD throughout the study, except on the 4^th^ occasion. SF and BIA differed on the 1^st^ and 2^nd^ occasion.

In boys, SF showed the strongest correlations with the underwater weighing method with r values ranging between .32 (non-significant) to .64 (p< .001). Only on occasions 1 and 2 body fat percentages as estimated by BIA and UWD correlated significantly. In girls, SF and BIA correlated comparably with the underwater weighing method, with r values ranging between .30 (non-significant) to .77 (p< .001).

### Longitudinal results

In both sex categories, only few subjects were measured before the age of PHV. On occasions one and two there were four boys who had not reached age at PHV. Three of them reached age at PHV before the third occasion and the last one reached age at PHV before the final occasion. Only one girl had not reached age at PHV on measurement occasions one and two. Therefore, the results before age at PHV should be interpreted with care.

In boys, PHV (10.1 ± 2.0 cm yr^-1^) occurred at the age of 13.0 ± 1.0 years. Girls reached their mean PHV (9.3 ± 3.7 cm yr^-1^) at the age of 11.6 ± 1.5 years, which was significantly earlier than in boys (p < .001). On average two years later linear growth came to an end in boys, whilst in girls a continuation in linear growth was observed until 4 years after age at PHV.

Table 3 presents the final model predicting body fat percentage in function of years from PHV for boys.

**Table 3:** Results of the final model for the development of body fat percentage according to years from PHV in boys.

**♂**	**b**	**SE**	**p**
**Intercept**	34.02	4.38	
**PHV**	-0.53	0.17	0.002
**Methods (ref = UWD)**			
**BIA**	1.04	0.76	0.17
**SF**	-0.17	0.70	0.81
Methods*PHV (ref = UWD)			
BIA*PHV	-0.57	0.20	0.004*
SF*PHV	0.18	0.17	0.29

SE: Standard Error; UWD: Underwater Densitometry; BIA: Bio Impedance Analysis, SF: Skinfold formula of Slaughter et al. (1988)

* significantly different from (α = 0.05). All analyses were adjusted for body height and weight.

In boys, body fat percentage estimated by UWD (b = -0.53, SE = 0.17, p = 0.002), BIA (b = -1.10, SE = 0.17, p< 0.001) and SF (b = -0.35, SE = 0.12, p = 0.003) significantly decreased with increasing years from PHV. For example, for body fat percentage estimated by UWD this means that each one-year increase in years from PHV is related to a decrease of 0.53% in body fat. However, the decrease in body fat percentage was significantly stronger for BIA estimations compared to UWD (interaction effect: b = -0.57, SE = 0.20, p = 0.004) and SF (interaction effect: b = -0.75, SE = 0.16, p< 0.001) estimations. As shown in Table 4, this difference in patterning resulted in significantly lower body fat percentage estimated by BIA compared to UWD and SF starting around four years from PHV. The decrease in body fat percentage observed by SF estimations did not significantly differ from the decrease observed by UWD estimations (interaction effect: b = 0.18, SE = 0.17, p = 0.29). UWD and SF gave comparable estimations of body fat percentage throughout all years from PHV measured.

**Table 4:** Predicted mean fat percentages according to years from PHV and measurement method for boys.

Years from PHV	UWD (SE)	BIA (SE)	SF (SE)
**-1**	10.3 (0.8) ^a^^,^^b^	12.0 (0.8) ^a^	10.0 (0.7) ^b^
**0**	9.8 (0.7) ^a^	10.9 (0.7) ^a^	9.6 (0.6) ^a^
**1**	9.3 (0.6) ^a^	9.8 (0.6) ^a^	9.3 (0.5) ^a^
**2**	8.8 (0.5) ^a^	8.6 (0.5) ^a^	8.9 (0.5) ^a^
**3**	8.2 (0.5) ^a^	7.5 (0.5) ^a^	8.6 (0.5) ^a^
**4**	7.7 (0.5) ^a^	6.4 (0.5) ^b^	8.2 (0.5) ^a^
**5**	7.2 (0.6) ^a^	5.3 (0.6) ^b^	7.9 (0.5) ^a^
**6**	6.6 (0.7) ^a^	4.2 (0.7) ^b^	7.5 (0.6) ^a^

PHV = Peak Height Velocity, SE = standard error

Predictions were calculated for a boy with an average height (175.0 cm) and weight (62.1 kg).

^a,b^ Within one row, means with the same indices do not differ significantly (α = 0.05).

Table 5 presents the final model predicting body fat percentage in function of years from PHV for girls.

**Table 5:** Results of the final model for the development of body fat percentage according to years from PHV in girls.

**♀**	**b**	**SE**	**p**
**Intercept**	41.7	9.1	
**PHV**	0.08	0.18	0.67
**Methods (ref = UWD)**			
**BIA**	4.12	1.17	<0.001*
**SF**	2.82	0.72	<0.001*
Methods*PHV (ref = UWD)			
BIA*PHV	-0.42	0.23	0.07
SF*PHV	-0.35	0.15	0.02*

SE: Standard Error; UWD: Underwater Densitometry; BIA: Bio Impedance Analysis, SF: Skinfold formula of Slaughter et al. (1988)

* significantly different from UWD (α = 0.05). All analyses were adjusted for body height and weight.

In girls, all 3 methods indicated a stable body fat percentage during the study period (UWD: b = 0.08, SE = 0.18, p = 0.67; BIA: b = -0.34, SE = 0.21, p = 0.11 and SF: b = -0.27, SE = 0.14, p = 0.05), but the pattern according to SF was significantly different from UWD (interaction effect: b = -0.35, SE = 0.15, p = 0.02). The patterns according to SF and BIA were similar (interaction effect: b = 0.06, SE = 0.19, p = 0.74). Table 6 shows body fat percentage in function of years from PHV. In comparison to UWD, BIA overestimated body fat percentage from one year before age at PHV until six years after, whilst SF gave an overestimation from one year before age at PHV until five years after. Moreover, BIA and SF gave significantly different values at three and five years after age at PHV.

**Table 6:** Predicted mean body fat percentages according to years from PHV and measurement method for girls.

Years from PHV	UWD (SE)	BIA (SE)	SF (SE)
**-1**	16.0 (1.3) ^a^	20.5 (1.6) ^b^	19.1 (1.2) ^b^
**0**	16.0 (1.2) ^a^	20.2 (1.4) ^b^	18.8 (1.1) ^b^
**1**	16.1 (1.1) ^a^	19.8 (1.3) ^b^	18.6 (1.0) ^b^
**2**	16.2 (1.0) ^a^	19.5 (1.2) ^b^	18.3 (1.0) ^b^
**3**	16.3 (1.0) ^a^	19.1 (1.1) ^b^	18.0 (0.9) ^c^
**4**	16.3 (1.0) ^a^	18.8 (1.0) ^b^	17.7 (0.9) ^b^
**5**	16.4 (1.0) ^a^	18.5 (1.0) ^b^	17.5 (0.9) ^c^
**6**	16.5 (1.0) ^a^	18.1 (1.1) ^b^	17.2 (1.0) ^a^^,^^b^

PHV = Peak Height Velocity, SE = standard error, UWD = Underwater Densitometry, BIA = Bio Impedance Analysis, SF = Skinfold formula of Slaughter et al. (1988)

Predictions were calculated for a girl with an average height (168.4 cm) and weight (55.0 kg).

^a,b,c^ Within one row, means with the same indices do not differ significantly (α = 0.05).

## Discussion

The present study combined cross-sectional with longitudinal analyses of body fat percentage in adolescent sprint athletes. Low to moderate correlations calculated on each occasion indicated important inconsistencies with UWD at the individual level in both sexes, but especially in boys for the BIA method.

In boys, cross-sectional analysis revealed no differences between UWD and both field methods, whilst BIA and SF differed mutually. Longitudinal analysis in function of years from age at PHV showed that, with UWD as the reference method, the SF based formula offers a better estimation of the development in relative body fatness as compared to BIA. BIA showed a different patterning resulting in an underestimation of body fat percentage starting from 4 years after age at PHV. In girls, both cross-sectional and longitudinal analyses showed that BIA as well as SF yielded significantly higher results than UWD throughout the study period, with BIA consistently delivering the highest overestimation.

According the ACSM position stand on Nutrition and Athletic Performance [14], the prediction accuracy of BIA is similar to SF thickness assessments with a preference for BIA because it does not require the technical skill associated with SF thickness measurements. Based on the present results, this cannot be supported, at least not in the case of adolescent sprint athletes. According to Loucks [15], skinfold equations to predict body fat percentage are preferred above BIA in athletic populations because of changes in hydration and glycogen stores. Another possible explanation for the deviating results of BIA from UWD may be the BIA formula used by the manufacturer. Although the ‘athletic’ mode was selected, this formula may not have been sufficiently specific for the current sample.

Research using cross-sectional designs has shown that the skinfold based formula of Slaughter et al. [7] can be advised for non-athletic males and females before, during and after puberty [16,17]. Wong et al. [16] concluded that this formula could be advised for adolescent females because of its accuracy and ease of practice, while Rodriguez et al. [17] found that the formula of Slaughter et al. [7] had the lowest error range from eleven skinfold based equations as compared to DXA. Based on the present study, this formula appears indeed to be sufficiently sensitive for changes in maturation in male adolescent sprint athletes, but it should be used with care when examining their female counterparts.

When interpreting the present results, some study limitations should be kept in mind. At first, in the present study, UWD was taken as the criterion or reference method. However, nowadays, UWD is no longer considered to be the “standard method”. Instead, imaging techniques such as DXA, RX and MRI are considered to be more precise [5]. Nevertheless, the cost of and access to these instruments make them difficult to use in a longitudinal study with a relatively high number of participants. Moreover, when using DXA or RX, the subject gets exposed to a certain radiation dose which is a reason for not using these methods too frequently. A second limitation was the use of a foot-to-foot BIA system, instead of the foot-to-hand system that allows to analyze a greater part of the body. Indeed, a study from Pietrobelli et al. [18] showed that an eight-electrode BIA system offers a more accurate estimation of body fat percentage than a conventional foot-to-foot BIA. Moreover, although it was attempted to standardize as much as possible with respect to clothing, hydration status and time of the day, this was not always possible for the latter. Finally, with a few exceptions, participants in this study appeared to have reached age at PHV already at the first measurement occasion. Therefore, our findings were less precise for years before PHV especially among girls (illustrated by the larger standard errors).

A strength of the present study is its longitudinal design, with six estimations of body fat percentage over a period of three years during which body composition is rapidly changing. Furthermore, original data in the critical period of adolescent growth were analyzed in function of years from PHV, taking into account the maturity status of the athletes. This allowed to evaluate the applicability of the selected field methods for the follow-up of body composition in the specific population of adolescent sprint athletes. By applying the multi-level model approach, longitudinal data of in total 35 male and 34 female adolescent sprint athletes were used in the analysis, including the 62% of subjects with missing data on one or more measurement occasions. With a traditional statistical approach using a two-way repeated measures ANOVA 62% of the subjects would have been excluded for analysis, resulting in a totally different outcome.

## Conclusions

Thus, based on the present results, using a novel statistical approach accounting for maturity status and comparing to UWD, the 1988 skinfold based formula of Slaughter et al. appears to be sufficiently sensitive to maturity related changes in fat mass and fat free mass in boys, but not in girls. Therefore, it should be the preferred field method above BIA in male adolescent sprint athletes.

In girls, development of relative body fatness as estimated by BIA and SF showed to be systematically higher than estimations from the UWD. Therefore, caution is needed when interpreting results yielded by these measurement methods since this may lead to unnecessary adaptations of the dietary and training regime. As is the case for skinfold based prediction equations, it is of utmost importance to follow standard measurement procedures and, if possible, to apply a population specific formula when using BIA. Further research is necessary to develop a valid and reliable field method to measure body fat in adolescent sprint athletes, especially in girls.

## Supporting Information

S1 File Original dataset. (XLS)
