# Correction: Estimating Body Composition in Adolescent Sprint Athletes: Comparison of Different Methods in a 3 Years Longitudinal Design

**DOI:** 10.1371/journal.pone.0223694

**Published:** 2019-10-03

**Authors:** The PLOS ONE Staff

## Notice of republication

An incorrect version of Supporting Information S1 File was published in error. The publisher apologizes for this error. This article was republished on September 26, 2019 to correct for this error. Please download this article again to view the correct version.
